# The Immune Characteristics of the Epididymis and the Immune Pathway of the Epididymitis Caused by Different Pathogens

**DOI:** 10.3389/fimmu.2020.02115

**Published:** 2020-09-30

**Authors:** Hu Zhao, Caiqian Yu, Chunyu He, Chunlei Mei, Aihua Liao, Donghui Huang

**Affiliations:** ^1^Department of Human Anatomy, Tongji Medical College, Huazhong University of Science and Technology, Wuhan, China; ^2^Institute of Reproduction Health Research, Tongji Medical College, Huazhong University of Science and Technology, Wuhan, China

**Keywords:** epididymis, epididymitis, epididymis epithelial cells, uropathogenic *Escherichia coli*, *Chlamydia trachomatis*, virus

## Abstract

The epididymis is an important male accessory sex organ where sperm motility and fertilization ability develop. When spermatozoa carrying foreign antigens enter the epididymis, the epididymis shows “immune privilege” to tolerate them. It is well-known that a tolerogenic environment exists in the caput epididymis, while pro-inflammatory circumstances prefer the cauda epididymis. This meticulously regulated immune environment not only protects spermatozoa from autoimmunity but also defends spermatozoa against pathogenic damage. Epididymitis is one of the common causes of male infertility. Up to 40% of patients suffer from permanent oligospermia or azoospermia. This is related to the immune characteristics of the epididymis itself. Moreover, epididymitis induced by different pathogenic microbial infections has different characteristics. This article elaborates on the distribution and immune response characteristics of epididymis immune cells, the role of epididymis epithelial cells (EECs), and the epididymis defense against different pathogenic infections (such as uropathogenic *Escherichia coli, Chlamydia trachomatis*, and viruses to provide therapeutic approaches for epididymitis and its subsequent fertility problems.

## Introduction

Infertility is the third major issue affecting human health, experienced by ~10–15% of couples when attempting to conceive a baby, among which 50% are related to male factor infertility (1). Infection and inflammation are involved in 13–15% of cases of male factor infertility. However, prevalence rates are up to 30% in regions with limited access to medical care (2, 3). Relevant diseases that can lead to infertility include epididymitis, combined epididymo-orchitis, and rarely, isolated orchitis (4, 5).

Acute epididymitis is one of the most common diseases related to male inflammation. Nicholson et al. have reported its incidence is ~2.5–6.5/100 000 person-years (5, 6). Epididymitis can occur in men of any age. Pilatz et al. (7) investigated 237 patients with acute epididymitis aged 18–97 years and found the highest incidence rate was in the group aged between 48 and 57 years old. Redshaw et al. (8) showed that the annual incidence of acute epididymitis is ~1.2/1,000 boys at the age of two to 13 years, while 43% of epididymitis cases occur among adult men aged 20–30 years. It is well-known that men with epididymitis usually present with impaired semen quality as well as a high number of white blood cells (4, 9, 10). Pilatz et al. (11) found the sperm protein composition changed significantly in patients after epididymitis, which may be one of the factors contributing to subfertility/infertility in men after episodes of epididymitis.

Although conservative antimicrobial therapy is possible in the majority of patients and is usually sufficient to eradicate the pathogen, studies have shown that up to 40% of patients suffer from permanent oligospermia or azoospermia (12). Rusz et al. reported that persistent detrimental effects are not uncommon in patients with acute epididymitis even after a complete bacteriological cure (9). Recent data showed that this was related to pathogen damage, strong fibrotic transformation, and epithelial degeneration (13). However, the research of immunopathological mechanisms related to human epididymitis is hindered due to the limited access to tissue samples (14). Therefore, studies on the epididymis have been largely performed in rats and mice to assess the morphological changes and immune pathways (12). Evidence shows that models mimicking epididymitis have been instructive in a better understanding of the mechanisms of disease initiation and progression (5).

This study describes the immune characteristics of the epididymis and the immune pathways of the epididymitis triggered by various pathogenic infections (*E. coli, Chlamydia trachomatis*, viruses, etc.) in animal models to explore how the immune related-mechanisms of epididymitis can impair male fertility.

## Immune Characteristics of the Epididymis

### Structure of the Epididymis

**Figure 1:**
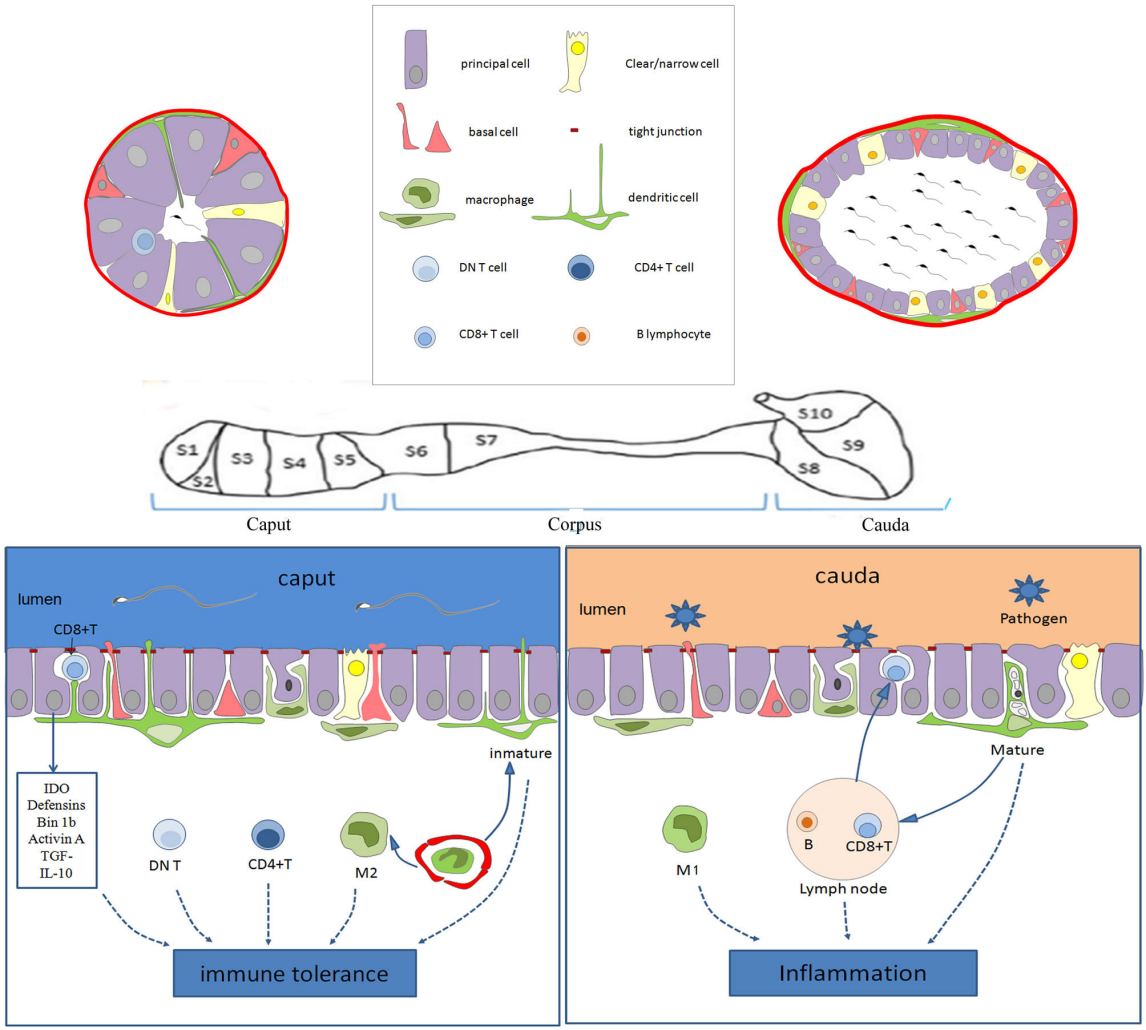
Schematic diagram showing the immune characteristics of the epididymis in the different regions. The epithelium of the epididymis contains various cell types, including the main cells, basal cells, lymphocyte cells, clear cells, dendritic cells, and macrophages. In the caput, dendritic cells could extend to the tight junction between the epithelial cells, and be particularly active. There are more CD4+ T cells and DN T cells in the caput than in the cauda. Moreover, the immune molecules (such as IDO, actin, defensins, and Bin 1b) are highly expressed in the caput, which together with immune cells provide an environment of the immune tolerance for the neo-antigens of spermatozoa. However, various pathogens coming into the genital tract always activate inflammation in the cauda epididymis. CD8+ T cells in the EECs and M1 macrophages facilitate the clearance of pathogens. Moreover, the B lymphocytes and lymphatics tend to increase toward the caudal region, increasing the ability to respond to ascending infections. IDO, tryptophan-catabolizing enzyme indoleamine 2,3-dioxygenase; DN T, CD4-CD8- T; EECs, Epididymis epithelial cells.

The epididymis is mainly composed of the epididymal tube. The inner layer of the epididymal duct is lined by a pseudostratified columnar ciliated epithelium, and the outer layer is surrounded by a peritubular layer of smooth muscle cells, in which there is interstitial tissue stroma containing the vasculature and lymphatics (15). Epididymis epithelial cells (EECs) are composed of many kinds of epithelial cells, including main cells, basal cells, lymphocyte or halo cells, clear cells, and monocyte phagocytes, among which the monocyte phagocytes include dendritic cells and macrophages (16), as shown in Figure 1.

The epididymis is divided into distinct segments according to its morphology and function (15). Usually, the epididymis is divided into three regions, including the caput, corpus, and cauda epididymis according to its anatomical structure (17, 18), but sometimes it is divided into four regions (the initial segment, caput, corpus, and cauda) (15, 19).

### Blood-Epididymis Barrier

The epididymis has a certain barrier function, which is called the Blood-Epididymis Barrier (BEB). The fully functional BEB consists of anatomical, physiological, and immunological barriers (20). The anatomical barrier is made up of tight junctions, formed from the principal cells' basolateral and apical membranes, which restrict molecules and cells from coming into or out of the lumen. The physiological barrier comprised of transporters and channels, while the immunological barrier is comprised of different immune components inside and outside the tubule/duct (20).

The tight junctions and selective transport by the principal cells can create a high concentration of some molecules in the epididymis, such as carnitine and inositol, which is helpful for sperm storage and maturing (21, 22). In addition to creating a suitable environment for sperm maturation, the BEB also provides an immune-privileged environment for sperm with neoantigens (23).

The BEB, together with the blood-testis barrier, plays a key role in preventing autoimmune responses against antigenic post-pubertal germ cells (20). Compared with the blood-testis barrier, the tight junctions of the epididymis are much less effective (24). Itoh et al. (25) demonstrated that spermatic granulomas were formed after injection of spermatozoa or testicular germ cells into the mice epididymal interstitial space; in contrast, no infiltrate was detected, and spermatic granulomas were not formed in the interstitial space when similar studies were performed in the testis. Alterations in the BEB may also bring about the formation of inflammatory conditions such as sperm granulomas (22). Infection and inflammation also damage the BEB, resulting in loss of the barrier function. Maintaining the integrity of the BEB is essential for sperm maturation and immune privilege (26). Loss of the barrier function opens a pathway for the entry of physiological and immunological components, which can alter the luminal microenvironment and lead to autoimmunity against sperm antigens, finally resulting in male infertility (27).

### EECs and TRLs

Toll-like receptors (TLRs) are innate immune pathogen pattern recognition receptors, which can recognize proteins, nucleic acids, and lipids of pathogenic microorganisms invading the body [such as viruses, bacteria, fungi, and protozoa (28)]. In rats, both EECs and immune cells express TLRs throughout the epididymis, which indicates that EECs also play an immune role (29). TLR1-TLR9 mRNA is abundantly expressed in the rat epididymis, while Tlr10 and Tlr11 are less abundantly expressed (30). However, the clear cells in the rat cauda epididymis do not express TLRs5–7 or TLR11 (29). TLRs1–4 are expressed by principal cells in all regions of the rat duct (30). The expression of TLR1-6 in the caput epididymis is similar to those in the testis, while the levels of TLR7, 9, and 11 in the mouse epididymis are higher (31).

## Immune Cells in the Epididymis

### Dendritic Cells

Dendritic cells (DCs) are “professional” antigen-presenting cells that internalize and process allo- and autoantigens (32). The DC maturation state is a key point for balancing tissue tolerance and autoimmunity. Immature DCs have a strong ability to internalize antigens, but their ability to activate T cells is low, while mature DCs is a T cell stimulant (33).

The maturation process of DCs includes a decrease in endocytic capacity and an increase in the major histocompatibility complex and chemokine receptors (34). Upon receiving an activated signal associated with inflammation, an immature DC will turn into a mature DC and then produce many inflammatory cytokines, such as tumor necrosis factor-alpha (TNF-α), interleukin (IL)-20, IL-23, and TNF-related apoptosis-inducing ligand (35). In addition, mature DCs will induce T-cell proliferation and polarize T cells toward Th17 and Th1 (36). The physiological role of epididymal DC is to regulate the complex interactions between immune tolerance and activation, which is a balance point in determining male fertility (34, 37).

Da Silva et al. (38) found that DCs in the mouse epididymis form a dense dendritic network in the basal area of the epididymal epithelium and are highly active within the proximal caput, with its protuberance extending through the epithelium. They also reported a previously unrecognized dense network of dendritic cells (CD11c+ CD103+ eDCs and CD11c+ CD103– eDCs) located at the base of the mouse epididymal epithelium, which displayed strong antigen-presenting and cross-presenting capabilities *in vitro*. DCs could collect antigens within the epididymal lumen and present them to the CD4+ T cells, thus regulating immunological reactions to spermatozoa and pathogens (39).

The conventional DCs in the murine epididymis are usually divided into cDC1 and cDC2 (40). The cDC1 type specifically deals with the cross-presentation of antigens (41), while cDC2 are mainly involved in T helper cell type responses (42). Voisin et al. (40) studied DC populations in the murine epididymis and found that cDCs1 account for 0.35 and 0.2% of the caput and cauda epididymis cells, respectively (*P* < 0.05) and that the cDC2 occupy 0.5% of the caput cells and 0.1% of the cauda cells (*P* < 0.001).

Wang et al. (37) showed that the number of CD11c+ DCs is relatively low in normal human epididymis unlike a dense network of CD11c+ DCs localized in the mouse epididymis. They concluded that the epididymis might have three phenotypically and functionally distinct subsets of DCs according to human and mouse data. They found that tolerogenic DC can: recognize normal sperm antigens; that immunogenic DCs detect and clear out abnormal sperm cells and exotic pathogens; and that inflammatory DCs: recruit Th17/Th1 cells (37).

### Macrophages

Macrophages are the main phagocytes in tissue and can be specified for the detection, phagocytosis, and destruction of bacteria and other harmful organisms. They can also present antigens to T cells and initiate inflammation by releasing cytokines. Macrophages can be subcategorized as classical (M1) and alternative (M2) macrophages (43). M1 are characterized by a very high expression of pro-inflammatory cytokines (IL-6, TNF-α, and IL-12) and the production of reactive oxygen species and nitric oxide, which facilitates the clearance of microbial pathogens (44). In contrast, M2 are defined by high secretion of anti-inflammatory cytokines [IL-10, transforming growth factor-β (TGF-β)] and low expression of pro-inflammatory cytokines (IL-12, TNFα) (45). Therefore, M1/M2 polarization could affect the fate of inflamed or injured organs (46).

Macrophages are the most abundant immunocompetent cells in the murine epididymis (47). Macrophages are located chiefly in the interstitial and peritubular regions of the epididymis. Macrophages in the interstitial regions of the epididymis could express major histocompatibility complex class II antigens, which are necessary for antigen presentation to T cells (48). In contrast, most macrophages in the peritubular regions of the epididymis lack major histocompatibility complex class II expression, which is a common feature of mucosal epithelia (49). Da Silva et al. (38) reported that a network of mononuclear phagocytes (MPs) expressing macrophage and dendritic cell markers such as CD11c, F4/80, and CX3CR1, lines the base of the mouse epididymal tubule. Both macrophages and DCs are “professional” antigen-presenting cells, and play a complex dual role in the epididymis. On the one hand, they could inhibit responses to sperm antigens under normal conditions, but on the other hand, they might activate inflammatory responses to pathogens during infection (16, 34).

### Lymphocyte

Lymphocytes present in the rat epididymal epithelium are often referred to as halo cells, which are named for the circular light-stained cytoplasm around the nucleus under light microscopy (50). Lymphocytes in the epididymis mainly consist of helper T lymphocytes (CD4+), cytotoxic T lymphocytes (CD8+), and B lymphocytes. CD4+ T cells are mainly located in the human epididymal stroma, while CD8+ T cells are the major lymphocytes within the epididymal epithelium (49). Voisin et al. used a single-cell isolation technique and observed that the numbers of CD4+ T cells were approximately equal in the murine caput and cauda epididymides (1.1 and 1%, respectively); the numbers of CD8+ T lymphocytes were similar as well between the caput and cauda (1.4 and 1.05%, respectively); however, the number of B lymphocytes in the cauda epididymis of was significantly higher than in the caput (0.7 and 0.35%, respectively) (40).

Surprisingly, natural killer and natural killer T cells were not detected in the murine epididymis (40). Given the very low incidence of epididymal tumors in humans (51), other populations or mechanisms may be involved in the anti-tumor effect in the epididymis (40). Voisin et al. (40) have described a new antigen-specific regulatory T cell population in the murine epididymis: double-negative (DN) T cells and found they were mainly located in the caput epididymis. This kind of cell has a strong cytotoxic effect on leukemia and lymphoma cell lines, but it does not affect normal cells. This supports the hypothesis that DN T cells could replace natural killer cells in the epididymis to prevent tumorigenesis (40).

Lymphocytes change in the epididymis at different ages (52). In young adult animals, the lymphocytes consist of helper T lymphocytes (CD4+) and cytotoxic T lymphocytes (CD8+), but there are no B lymphocytes, which indicate that immunoglobulin is not produced in the epididymis under normal conditions (52). A study of old brown Norwegian rats showed that B lymphocytes were rare in the epididymis epithelium of young rats, fewer than 1% of the total number of immune cells, but they were occasionally found in the epididymis epithelium of older rats, accounting for ~5% of the total number of immune cells (53). The author postulated that the accumulation of damaged epithelial cells and antigens of germ cell origin, leaking through a dysfunctional blood-epididymis barrier, might contribute to the active recruitment of immune cells with age (53).

### Basal Cell

Basal cells (BCs) are present in the epididymal epithelium of all mammalian species and are located in the basal region. Several manuscripts have reported BCs in the epididymis are similar to peritubular macrophages in their ultrastructural and antigenic aspects, and presumed that epididymal BCs from primates and rodents might originate from circulating progenitors and play some immunological role (54, 55). However, a recent article reported that mouse BCs have similar morphological features compared to those of adjacent epithelial cells, and the authors proposed that BCs originate from the non-differentiated columnar epithelial cells instead of the MP system (56). Mammalian BCs not only have a hypothesized scavenger function but are also regarded as luminal sensors to regulate the activity of principal and clear cells (57).

### Other Cells

The γδ T cells are typical mucosal cells and have recently been identified in the stroma and epididymal epithelium of the murine epididymis (40). The γδ T cells account for only a minor population of the T cells in the peripheral blood and lymphoid tissues in both mice and humans, but they are abundant in epithelial tissue (58). Epithelial γδ T cells exhibit tissue-specific restricted TCRs and show innate-like properties (59). Daley et al. (60) found that γδT cells could disable the immune system response against human pancreatic ductal adenocarcinoma. Considering the characteristics of the epididymis, γδT cells could also disable the immune system against sperm.

## Innate Immune Molecules

### Indoleamine 2,3-Dioxygenase

Indoleamine 2,3-dioxygenase (IDO) is an intracellular enzyme that catalyzes tryptophan to N-formalkynurenine, which is the first and key step in the kynurenine pathway (61). IDO is a ubiquitously expressed cytoplasmic protein typically activated by interferons (IFNs) (62). IDO has two main functions: one is to deplete tryptophan in an enclosed environment (such as in the epididymal duct lumen) to prevent bacterial or viral infection, and the other is to suppress T-cell-mediated immune responses against self-antigens, fetal antigens, or allogeneic antigens (63). IDO has been proven to suppress many kinds of immune responses, such as the regulation of tissue tolerance and the maintenance of immune privilege (64, 65).

Britan et al. (66) reported that IDO is highly expressed in the mouse caput epididymis and is mainly secreted by the principal and apical cells. The drosophila mothers against the decapentaplegic protein 2/3/4 signaling pathway activated by activin A, and could regulate the expression of IDO, suggesting that activin A may be an activator of IDO expression in the mouse proximal epididymis (67). Moreover, these immune regulators may induce a tolerogenic circumstance in DCs and T cells (67, 68). Jrad-Lamine et al. (69) have shown that pro-inflammatory cytokine expression is increased in the caput epididymitis of IDO-deficient mice. IDO-deficient mice possess significantly greater numbers of morphologically defective spermatozoa compared with wide-type controls. We propose that IDO activity, on the one hand, controlled the level of epididymal leukocytes and, on the other, regulated the involvement of the epididymal proteasome in clearing defective transiting spermatozoa (69).

### Defensins

Defensins are not only important regulatory molecules in the biological immune system but are also important antimicrobial peptides with direct bactericidal functions (70). Multiple beta-defensins have been found in the epididymis of humans and mice with region-specific expression patterns, showing bacteria-killing activity and the promotion of sperm motility (71, 72).

Bin1b, exclusively expressed in the rat caput region of the epididymis, is a natural epididymis-specific antimicrobial peptide and can promote immature sperm to obtain motility (73). The specific expression of β-defensin 126 in the cattle epididymal tail can promote the acquisition of motility of epididymal sperm (74). Yenugu et al. (75) found that human and macaque sperm associated antigen 11 is involved in the mechanism of epididymal sperm maturation and host defense. Human β-defensin 114 can regulate lipopolysaccharide mediated inflammation and prevent the loss of sperm motility (76). Lack of human β-defensin 1 can lead to male infertility, which is manifested by poor sperm motility and reproductive tract infections (77).

### Other Immune Molecules

The TGF-β superfamily comprises more than 30 members, including TGF-β isoforms, bone morphogenetic proteins, growth and differentiation factors, activins/inhibins, NODAL, and the anti-Müllerian hormone (78). Three isoforms of TGF-β have been identified in mammals: TGF-β1, TGF-β2, and TGF-β3. A recent study has revealed that TGF-β signaling in DCs is required for immunotolerance to sperm located in the epididymis, and that male mice lacking TGFβ signaling in DCs would develop severe epididymal inflammation (34). Voisin et al. (79) showed that EECs expressed the three TGF-β isoforms in all regions of the murine epididymis in pre-pubertal to adult mice, which indicated that the epididymal epithelium plays an active role in establishing a pro-tolerogenic environment necessary for the survival of immunogenic spermatozoa.

Activin A, a member of the TGF-β superfamily, is an important regulator of testicular and epididymal development and function, as well as inflammation and immunity (67). In the adult murine reproductive tract, activin A mRNA levels are highest in the caput epididymis and decrease progressively toward the distal vas deferens (80, 81). IL-10, a key effector regulatory cytokine, is located in the principal cells of the murine epididymal epithelium and may be involved in the protection of spermatozoa from autoimmune reactions (82). There are many other antimicrobial proteins (such as cathelicidins, mucins, lactoferrin, and chemokines) involved in the innate defense of EECs (83, 84).

## Characteristics of Epididymis Immunity

In contrast to the immunologically privileged testis, the epididymis does not support prolonged allogeneic graft survival (85). Furthermore, epithelial tight junctions of the rodent epididymis may not be as effective as those of the blood-testis barrier, and direct interactions between intra-epithelial immune cells and either sperm antigens or ascending pathogens is possible (71). Therefore, the epididymis is more prone to inflammation and autoimmune responses than the testis.

It is worth noting that the type and number of immune cells and innate immune molecules are most abundant in the murine epididymis head (40, 72). However, the distribution of epididymal lymphatics tends to increase toward the murine caudal region (86). It is widely accepted that immune tolerance exists in the caput epididymis, but pro-inflammatory circumstances prefer the cauda epididymis, as shown in Figure 1. Moreover, the closer the epididymis is to the testis, the lower the probability of inflammation, and the farther the testis is from the epididymis, the greater the odds of inflammation and its deleterious effect on reproduction (87). Epididymal immunity is based on a finely tuned equilibrium between efficient immune responses to pathogens and strong tolerance to spermatozoa (83).

## Immune Pathways of the Epididymitis Caused by Different Pathogens

Although epididymitis can occur in men of any age, the majority of epididymitis cases occur in men aged 20–39 and they are most often associated with sexually transmitted diseases (5). *Chlamydia trachomatis* and *Neisseria gonorrhea* account for ~50% of cases of epididymitis associated with chlamydia and gonorrhea in men <39 years of age. After 39 years of age, the most common aetiologic agent responsible for epididymitis is *Escherichia coli* and other coliform bacteria found in the gastrointestinal tract (88). Weidner et al. (89) reported that enteric pathogens mainly occur in patients older than 35 years, sexually transmitted pathogens like Chlamydia trachomatis and Neisseria gonorrhoeae are often responsible in patients under 35 years. The different pathogens have different susceptible populations, and they have different inflammatory immune mechanisms. Therefore, it is helpful to understand the immune response of various pathogens for the treatment of epididymitis.

## Bacterial Infection (Uropathogenic *Escherichia Coli*)

Uropathogenic *Escherichia coli* (UPEC) is one of the most common causes of acute epididymitis, which is usually due to the retrograde progression of urethral pathogens and sexually transmitted bacterial infections (90). Silva et al. (91) reported that experimental epididymitis induced in rats by Gram-negative (LPS) and Gram-positive bacterial products resulted in differential patterns of acute inflammation in the cauda epididymis. LPS elicited a strong inflammatory reaction, as reflected by the upregulation of the levels of mRNA for seven inflammatory mediators (IL-1b, TNF, IL-6, Interferon-gamma, IL-10, nitric oxide synthase 2, and nuclear factor-kappa-B inhibitor alpha), and the tissue concentrations of six cytokines/chemokines (IL-1a, IL-1b, IL-6, IL-10, CXCL2, and CCL2) within the first 24 h post-treatment (91).

**Figure 2:**
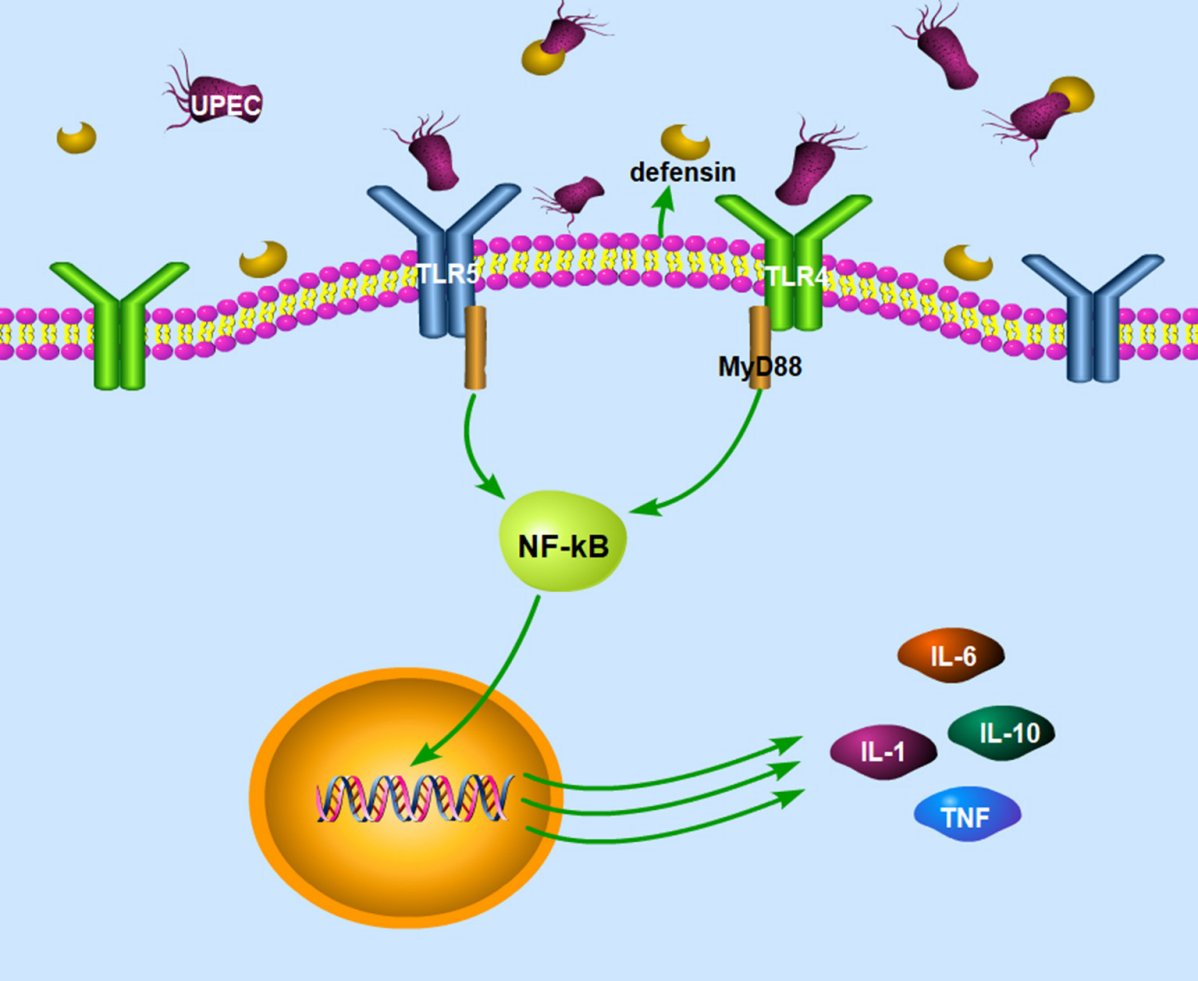
The signaling pathways in the epididymitis induced by UEPC. The signaling pathway mainly includes two parts: (1) UPEC could activate TLR4 and TLR5 on epithelial cells and macrophages and induce the production of pro-inflammatory cytokines through the classical inflammatory NF-kB signaling pathway. (2) Epididymal cells can also limit pathogen infections by secreting defensins after infection with UPEC. UPEC, uropathogenic *Escherichia coli*; TLR4, toll like receptor 4; TLR5, toll like receptor 5; MyD 88, myeloid differentiation factor 88; NF-kB, nuclear factor kappa B; IL-1, interleukin-1; IL-6, interleukin-6; IL-10, interleukin-10; TNF, tumor necrosis factor.

Cheng et al. (92) found that the mouse epididymis infected by *E. coli* could activate TLR4 and TLR5 in the epididymis head epithelial cells and macrophages and induce the production of pro-inflammatory cytokines through the classical inflammatory NF-kappa B signaling pathway. Meanwhile, the natural immune response induced by UPEC in the epididymis of TLR4 and TLR5 knockout mice (TLR4^−/−^, TLR5^−/−^) was significantly lower than that induced in the wild-type mice (92), as shown in Figure 2. Although UPEC also activates TLR11, TLR11 cannot initiate innate immune responses in humans because there is no functional TLR11 (93). Moreover, UPEC induced the production of type 1 interferons by EECs through the activation of interferon regulatory factor 3 (92).

Epididymal cells can also limit pathogen infection by secreting defensins after infection with UPEC in the mouse model (34). Gene expression of defensin b2, defensin b21, and defensin 27 in the caput epididymis decreased in the LPS-induced rat epididymitis (94), while the gene expression of defensin b29, defensin b41, and defensin b42 remained unchanged after the treatment (95). Biswas et al. (96) demonstrated that the expression of defensins and sperm associated antigen 11 genes increased in the epididymis and testes in a UPEC induced rat epididymo-orchitis model. The recombinant defensin 21 significantly decreased the bacterial load in the epididymis and testis and proved to be more effective than gentamycin. Moreover, overexpression of Bin1b helps mice to resist *E. coli*-induced epididymitis (97).

Epididymitis also results in long-term problems in patients, such as the irreversible development of ductal obstructions and fibrotic tissue remodeling, even if full antibiotic treatment is provided (98). In the chronic UEPC murine epididymitis, the main pathological changes are dominated by lymphocytes, plasma cells, interstitial fibrosis (99), and even dead UEPC were able to disrupt testicular architecture after epididymal injection in the rat model (100). However, the mechanisms for this are unclear and need to be characterized in the future.

## Immune Response Induced by *Chlamydia Trachomatis*

*Chlamydia trachomatis* (CT) is the most common sexually transmitted pathogen in high-income countries (101, 102) and is the most common etiology of STIs among sexually active males 14 to 35 years of age (88). Ito et al. (103) examined different microorganisms in men younger than 40 years of age with acute epididymitis and found that CT is a major pathogen (28%) while the prevalence of genital mycoplasmas (5%) and ureaplasmas were lower (5%). Screening for CT is advocated to control the transmission of chlamydia in sexually active young adults (104). However, there is an opposing option posited by another study, in which a population-based test for urogenital C trachomatis infection did not reduce the long-term risk of reproductive complications in women or epididymitis in men (105).

**Figure 3:**
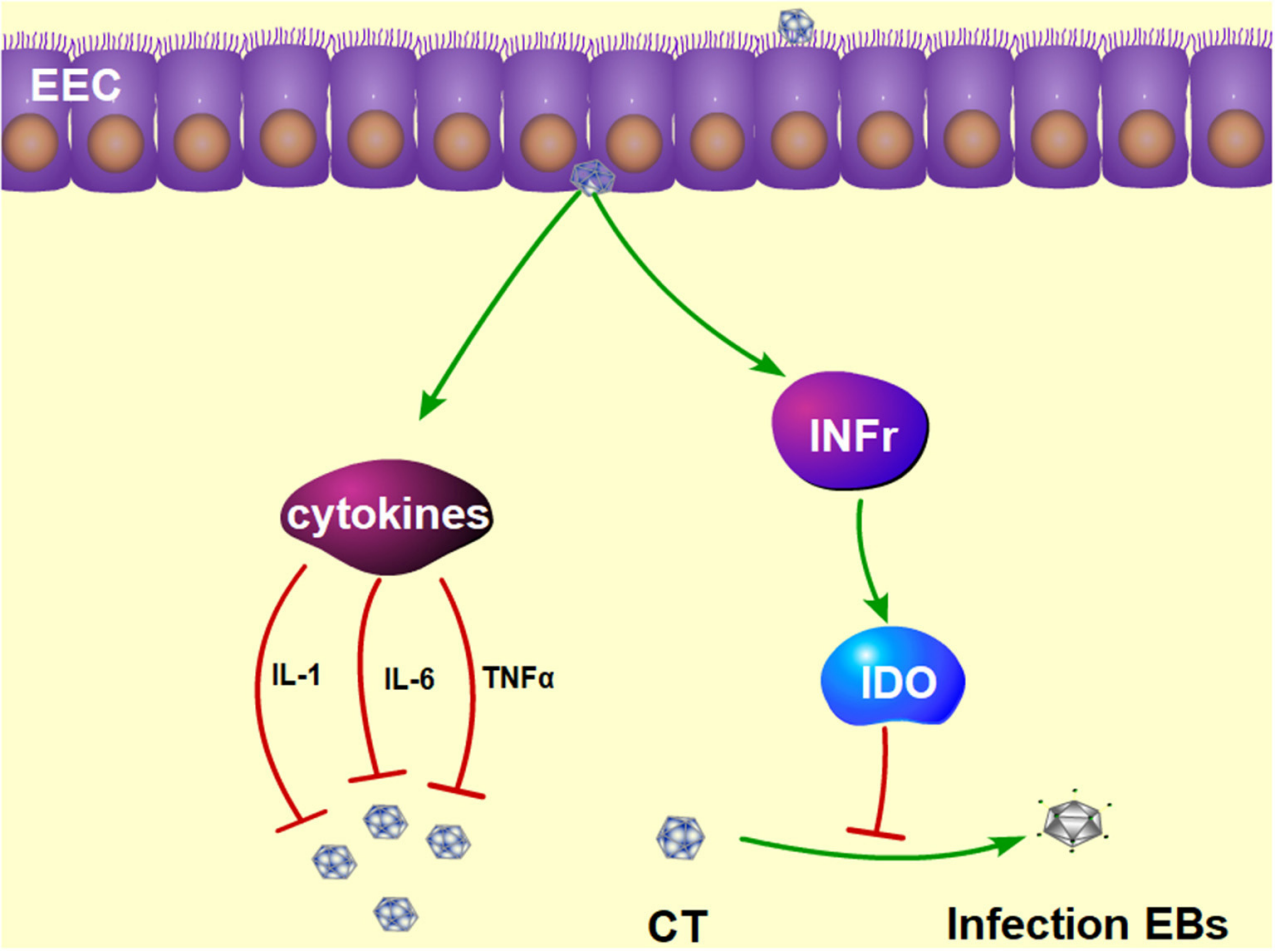
The protective mechanism against CT infection in the epididymis. Interferon γ plays a functional role in inhibiting the growth of CT by induction of IDO, which prevents the CT from differentiating into infectious EBs. Furthermore, CT infection can induce the production of pro-inflammatory cytokines, such as IL-1, Il-6, and TNFα. IDO, tryptophan-catabolizing enzyme indoleamine 2,3-dioxygenase; EBs, elementary bodies; IL-1, interleukin-1; IL-6, interleukin-6; TNFα, tumor necrosis factor alpha.

Approximately 50% of CT infections in men are asymptomatic (106, 107). The most important profile of a CT infection is a local immune response. First, the immune cells are recruited to the site of the infection and secrete pro-inflammatory cytokines such as interferon-γ (IFN-γ) (108, 109). IFN-γ has a dual function of inhibiting the growth of CT (110) in mice and inducing Th1 immune responses in CT-infected women (111). Studies have shown that IFN-γ activates host cells to restrict intracellular growth of CT by induction of IDO (112), as shown in Figure 3. The depletion of tryptophan by IDO has been proved to deprive CT of 5-hydroxytryptophan, which is essential for its differentiation into an infectious elementary body (112, 113). If IFN-γ is removed from the host cell, tryptophan synthesis will subsequently recover, and CT quickly forms into an infectious elementary body (114). In addition, CT infection of murine epithelial cells can induce the secretion of pro-inflammatory cytokines such as IL-1, Il-6, and TNF-α (115).

The most important features of CT infection are its chronic nature and the presence of a mild inflammation that remains subclinical in most individuals (116). Recognition of CT antigens is associated with TLR2, TLR4, and other CT antigens and pathogen recognition receptors (PRRs), which will induce a local secretion of cytokines/chemokines and consequently provoke chronic inflammation (116). This chronic inflammation can lead to cell proliferation, tissue remodeling, and scarring in the male genital tract (108).

## Induction of Antiviral Immune Responses in Epididymal Epithelial Cells

Viruses can be divided into DNA viruses and RNA viruses. DNA viruses that can infect the male reproductive system include adeno-associated virus, cytomegalovirus, herpes simplex virus (HSV), hepatitis B virus, human papillomavirus, etc. (117). RNA viruses include mumps virus, enterovirus (enterovirus), human immunodeficiency virus (HIV), hepatitis C virus, etc. (118).

Mumps virus is the most common RNA virus that causes viral orchitis. The orchitis caused by Mumps virus is usually accompanied by viral epididymitis, and it severely impairs male fertility (119). Park et al. (120) investigated 18 patients with mumps orchitis and found 13 patients (72.2%) were accompanied with epididymitis. HSV-2 is the most common DNA virus that infects the male genitals, but most patients infected with HSV-2 have no obvious symptoms (121). The epididymis is a major target and reservoir of HIV (122). Pilatz et al. (11) investigated the etiology of acute epididymitis and found that viral epididymitis seems a rare condition.

Viral infections of the epididymis may sexually spread pathogens (123, 124). Different virus types that can infect the epididymis are associated with male infertility (123). Therefore, understanding the mechanisms underlying epididymal innate antiviral responses would aid in the development of preventive and therapeutic strategies against viral infections of the epididymis (58).

The mouse EECs abundantly express viral sensors TLR3, the retinoic acid-inducible gene I (RIG-I), and DAI (31). TLR3 and retinoic acid-inducible gene I in EECs can be activated by synthetic double stranded RNA polyinosinic-polycytidylic acid, then significantly induce the expression of inflammatory cytokines (TNF-α and Monocyte chemokine-1, IFN-α and IFN-β) in EECs; the signaling pathway of DNA sensors can be initiated by HSV60 (31). HSV60 can significantly increase the expression of IFN-β, but not TNF-α and Monocyte chemokine-1 (31). Brown et al. (125) also showed that TLR3 and retinoic acid-inducible gene I-like receptors are enriched in human EECs from the corpus and cauda regions. Moreover, paired box 2, which was implicated in regulating antiviral response pathways, is required for basal expression of the DNA sensor, Z-DNA binding protein, and type I interferon, in caput but not in cauda cells (125).

## Conclusion

Epididymitis is one of the common causes of male infertility. Persistent detrimental effects are common even after a complete bacteriological cure. This is related to the immune characteristics of the epididymis itself. Here, we elaborated on the distribution and role of the epididymis immune cells and the mechanisms of epididymis defenses against different pathogenic microbial infections. The purpose of this manuscript is to provide guidance for understanding the immune environment of the epididymis and to guide future therapeutic approaches to treating epididymitis. However, human data on different causes of acute epididymitis is spare, so more research is needed in the future.

## Author Contributions

HZ and CY wrote the article and performed literature searches and data compilation. CH and CM performed the necessary literature searches and data compilation. AL revised the article and gave valuable suggestions. DH designed the review and checked and approved the submitted manuscript. All authors have read and approved the final manuscript.

## Conflict of Interest

The authors declare that the research was conducted in the absence of any commercial or financial relationships that could be construed as a potential conflict of interest.
